# Subdividing BI-RADS category 4 breast lesions observed on magnetic resonance imaging: Is it feasible?

**DOI:** 10.1590/0100-3984.2016.49.3e1

**Published:** 2016

**Authors:** Almir Galvão Vieira Bitencourt

**Affiliations:** 1 PhD, Graduate Program Advisor at A.C.Camargo Cancer Center, São Paulo, SP, Brazil. E-mail: almir.bitencourt@accamargo.org.br.

In clinical practice, there are various purposes for which magnetic resonance imaging (MRI) of the breasts is indicated, from the screening of high-risk patients to the staging and treatment planning for patients with breast cancer. This method has better sensitivity than conventional imaging (mammography and ultrasound) for the diagnosis of malignant breast lesions and has greater accuracy in evaluating the size and morphological features of tumors, as well as in detecting multifocal and multicentric lesions. However, despite the high sensitivity of MRI, many studies have reported that its specificity is low and that it produces a large number of false positives, which can lead to unnecessary biopsies and surgical procedures.

The Breast Imaging Reporting and Data System (BI-RADS), developed by the American College of Radiology and continually updated since 1992, is a guide with recommendations for the standardization of breast imaging (mammography, ultrasound, and MRI) reports and for the auditing of centers employing such methods^(**1****-****3**)^. Its objective is to standardize the nomenclature used in the reports, which should have a diagnostic conclusion and should propose management, according to the probability of malignancy. However, the cases classified as suspicious (BI-RADS category 4) show wide variation in the risk of malignancy (2-95%), which led to the subdivision of this category, as follows: 4A (low suspicion, risk of 2-10%); 4B (intermediate suspicion, risk of 11-50%); and 4C (high suspicion, risk of 51-95%). In the most recent editions of the BI-RADS, this subdivision was incorporated into the lexicon of mammography and ultrasound, although it has yet to be incorporated into that of MRI, because there is a lack of published studies to support such assessment^(**4**)^.

Published in this issue of **Radiologia Brasileira**, the article "Predictive performance of BI-RADS magnetic resonance imaging descriptors in the context of suspicious (category 4) findings" is one of the first in the literature to assess the likelihood of malignancy related to MRI findings in lesions classified as BI-RADS category 4^(**5**)^. In that study, Almeida et al.^(**5**)^ present consistent methodology and statistical analysis, emphasizing the credibility of their findings. This type of study is essential to defining the criteria to be used for the subdivision of suspicious findings into the categories 4A, 4B, and 4C. This subdivision can be even more important in MRI, in order to identify the need for a histological diagnosis in cases in which the lesions are not characterized by the conventional methods, because MRI-guided biopsy is a procedure that has a high cost and limited availability in Brazil. In addition, knowledge of the likelihood of malignancy in suspicious MRI findings can facilitate the correlation between the radiological and pathological findings, suggesting the need for further investigation by surgical resection of the lesions in which the histopathological results of a percutaneous biopsy are discordant.

The incorporation of functional sequences, such as diffusion and spectroscopy, can further contribute to the evaluation of suspicious findings in the morphological and dynamic assessments that are already part of the routine in MRI of the breasts^(**6**)^. With the growing number of studies related to the topic, it is likely that these methods will be incorporated into future editions of the BI-RADS. Recently, Almeida et al.^(**7**)^ published a study in the **American Journal of Roentgenology** showing how diffusion, a sequence that evaluates the movement of water molecules in tissues, can also contribute to the subdivision of BI-RADS category 4 breast lesions^(**7**)^.

The Almeida et al.^(**5**)^ article provides a greater understanding of MRI in patients with suspicious breast lesions, demonstrating that the presence of certain findings can increase the risk of malignancy in such patients. These results highlight the feasibility of subdividing BI-RADS category 4 lesions, which will provide more accurate diagnoses and allow individualized management.
